# Multimodal system for automated medical documentation and clinical decision support integrating contact center solutions

**DOI:** 10.1038/s41598-026-45879-2

**Published:** 2026-03-26

**Authors:** Małgorzata Płaza, Mirosław Płaza, Małgorzata Lucińska, Michał Młodawski, Justyna Kęczkowska, Stanisław Deniziak, Tamara Murawska, Kamil Murawski, Karol Wykrota, Dawid Jaszczyk, Michał Zawadzki, Marcin Kozłowski, Arkadiusz Gajda, Karol Twardowski, Zbigniew Koruba

**Affiliations:** 1 Kielce University of Technology, Al. Tysiąclecia P.P. 7, 25-314 Kielce, Poland; 2 Altar Sp. z o. o., ul. Różana 5, 25-729 Kielce, Poland

**Keywords:** Medical documentation, Semantic analysis, Expert system, Contact center systems, Computational biology and bioinformatics, Health care, Mathematics and computing, Medical research

## Abstract

The article presents the Parrot AI smart system, which supports physicians specialising in internal medicine and pediatrics. The solution is designed to automate time-consuming administrative processes and assist physicians in clinical decision-making. The main purpose of the system is to relieve medical staff of administrative tasks, such as manually filling out medical records, which translates into increased work efficiency and more time to focus on direct contact with patients. Parrot AI uses advanced natural language processing methods and generative language models to enable automated semantic analysis of medical interviews. Based on this, the system generates the content necessary to complete the electronic medical records and assists the physician by suggesting preliminary diagnoses and treatment options. Interaction with the patient can take place both at the initial medical interview stage (via chatbot or voicebot) and during a personal appointment at the clinic. Functional tests have demonstrated that the solution is highly effective. All tested components of the system achieved results well above the 80% effectiveness threshold, confirming the validity of further implementing the system into everyday clinical practice. Parrot AI has the potential not only to streamline physicians’ work, but also to improve the quality and consistency of medical records.

## Introduction

The rapid development of technologies based on artificial intelligence (AI) is currently one of the key challenges in numerous areas of social, economic, and technological life. The healthcare system is one area where AI-based solutions can significantly improve the quality of patient care. Most AI applications in medicine currently focus on developing specialised systems that support medical processes such as diagnostic imaging^1–4^, data analysis^5,6^, decision-making^7–9^, and clinical trials^10,11^. Additionally, the integration of components from Call/Contact Center (CC) systems (an area that has also seen significant technological advancement in recent years^12–15^ offers promising avenues for optimising healthcare operations and patient communication. Table 1 lists examples of commercial systems that use technological solutions to support physicians at work.

**Table 1:** Selected commercial systems using AI in medicine.

Name	Description	Functionality	References
*Global solutions*			
Nabla	AI assistant for medical records	Patient consultation transcription, documentation summaries, integration with electronic medical records	^16^
MedRespond	Educational platform	Patient education, automated question answering, telemedicine support	^17^
Infermedica	Diagnostic support system	Patient symptom analysis and suggestions for possible causes	^18^
Nuance Dragon Medical One	Cloud-based solution for medical records	Speech recognition, integration with electronic medical record systems	^19^
IBM Watson Health	AI-based platform for diagnostic support	Medical data analysis, clinical decision-making support, integration with medical records	^20^
Epic Systems	Supplier of medical records systems	Electronic medical records, patient data management, integration with other systems, support for telemedicine	^20^
*Solutions on the Polish market*			
Noa Notes (ZnanyLekarz Sp. z o.o.)	AI assistant to support physicians in creating medical records	Automatic transcription of physician-patient conversations in real time, generation of consultation summaries, integration with the ZnanyLekarz.pl platform	^21^
Previsit AI	AI system to assist physicians in collecting information from patients before the appointment	Collecting information from patients before the appointment, generating preliminary reports	^22^
Talkie.ai	Virtual registrar — medical voicebot	Automatic registration and cancellation of appointments, reminders for scheduled appointments, integration with electronic medical record systems	^23^
ADMEDVOICE	Intelligent speech processing system	Speech processing, recognition of medical terminology, structuring of test results, supporting the therapeutic process	^24^

Literature reviews indicate that there is currently a lack of dedicated, comprehensive, and multimodal solutions that directly support the work of general practitioners. Currently, despite significant progress in digitising healthcare, much of the data for electronic health records (EHR) is entered manually^25^. Doctors or dedicated staff usually fill out the necessary forms manually during or immediately after the medical interview, which is estimated to take approximately 49.2% of the entire appointment^27^. These processes significantly increase the length of appointments and heavily burden physicians with additional duties^27,28^. Solving these problems would reduce the time physicians spend on routine administrative tasks, allowing them to focus more on patient care. These issues and shortcomings motivated the authors to undertake work in this area and conduct research on data gathered in Poland. According to data from the Statistics Poland^27^, 181.1 million consultations were provided in Poland as part of primary health care in 2023 (an increase of 2.8% compared to 2022). These data indicate the ongoing need to automate patient care processes to help primary care physicians.

One of the stages involved in automating medical documentation processes (filling out forms) is ASR (Automatic Speech Recognition). Research on ASR systems is currently being conducted on a wide scale^30,31^. The primary way to assist physicians in automatically completing the first part of the medical forms – documenting symptoms and medications – may be through a solution based on semantic analysis of the patient interview. A dedicated prediction method could further help physicians complete the second part of the form, which typically includes the diagnosis and recommended treatment. Additional support for form automation may be achieved by integrating CC solutions, such as voice- and text-based virtual assistants, into the system. Their task would be to carry out the standard registration process and, above all, to conduct an initial interview, which would allow the physician to access the initial data before the actual appointment.

In view of the above, this paper contributes to the body of knowledge as follows:

- Development of a new smart, multimodal, and hybrid IT system that solves problems related to the automation of medical form filling processes. The proposed approach combines: (1) semantic analysis methods using ASR methods, NLP techniques, and generative AI/ML models to extract information from medical interviews, (2) methods supporting the use of knowledge and facilitating decision-making implemented in the form of an expert system, (3) components of call/contact centre systems in the form of virtual assistants supporting both voice and text communication channels.
- Conducting research to determine the effectiveness of individual components of the system, along with the selection of optimal parameters for these methods.

To the best of the authors’ knowledge, the proposed approach is the first solution to the problem of comprehensive automation of EHR completion processes by general practitioners specialising in internal medicine and pediatrics, thereby reducing the administrative burden on physicians and ultimately shortening the duration of appointments. The solution that has been developed is called Parrot AI.

.

## Materials and methods

The development of the proposed Parrot AI system included the following stages of research work:

- collection of representative data sets comprising anonymised actual interviews of patients with primary care physicians, as well as preparation of training sets for the knowledge base used by the expert system,
- transcription and labeling of recorded transcripts,
- development of a proprietary method for automating the process of obtaining information from medical interviews,
- development of an expert system used in diagnosis and therapy prediction,

development and integration of CC system components.

### Data

The first step was to collect recordings of medical interviews conducted by an internist and a pediatrician, which were anonymised in accordance with the requirements of the General Data Protection Regulation (GDPR). The first source was therefore recordings and transcripts of medical interviews (Table 2). The data examined includes a total of 2,000 recordings of conversations between physicians and patients, together with their transcripts obtained automatically and manually. All data were actual interviews conducted in Polish directly in the physician’s office. The databases prepared in the further part of the article are marked as: DBMI1, DBMI2, DBT1, DBT2.

**Table 2:** Database of interview recordings and transcripts.

Name	Description	Application
DBMI1	900 recordings of real internal medicine interviews ranging from 3 min 1 s to 31 min 30 s in length. The total duration of all recordings is 153 h, 51 min, and 27 s	The recordings were necessary for the transcription processes
DBMI2	1100 recordings of real pediatric medicine interviews ranging from 3 min 2 s to 28 min 52 s in length. The total duration of all recordings is 180 h, 31 min, and 47 s	The recordings were necessary for the transcription processes
DBT1	A set of transcripts of conversations obtained through automatic and manual transcription for the DBMI1 set	Data used in labeling, learning, and testing processes during the implementation of the automatic form filling module in the field of internal medicine
DBT2	A set of transcripts of conversations obtained through automatic and manual transcription for the DBMI2 set	Data used in labeling, learning, and testing processes during the implementation of the automatic form filling module in the field of pediatrics

The second step involved preparing databases containing synthetically generated data, which were used to develop a knowledge base dedicated to the expert system (Table 3). The databases created in this regard are marked as: DBSE1, DBSE2, DBSE3.

**Table 3:** Databases used for expert system.

Name	Description	Application
DBSE1	A teaching set comprising 1,235 cases of various symptom groups occurring in individual diseases in the field of internal medicine. The set includes 330 labels for 67 different diseases according to the ICD-10 classification	Data used for the learning processes of models developed for internal medicine
DBSE2	A teaching set comprising 5,726 cases of various symptom groups occurring in individual diseases in the field of pediatrics. The set includes 295 labels for 38 different diseases according to the ICD-10 classification	Data used for the learning processes of models developed for pediatrics
DBSE3	A set containing structured data with metadata and hierarchical relationships of ICD-10 codes	Data used to categorize, group and interpret medical cases

The collected data was supplemented with additional data sets collected as part of the integrated components of the CC system (voicebot and chatbot), which handled the automatic appointment registration process and collected data from the initial medical interview(Table 4). The prepared databases were labeled as: DBVB1, DBVB2, DBCB1, DBCB2.

**Table 4:** Database compiled as a result of the CC system.

Name	Description	Application
DBVB1	64 conversation recordings with voicebot component for internal medicine	The recordings were necessary for the transcription processes
DBVB2	61 conversation recordings with voicebot component for pediatrics	The recordings were necessary for the transcription processes
DBVT1	A set of transcripts of conversations obtained through automatic and manual transcription for the DBVB1 set	Data used in labeling, learning, and testing processes during the implementation of the automatic form filling module in the field of internal medicine
DBVT2	A set of transcripts of conversations obtained through automatic and manual transcription for the DBVB2 set	Data used in labeling, learning, and testing processes during the implementation of the automatic form filling module in the field of pediatrics
DBCB1	76 text conversations with chatbot component for internal medicine	Data used in labeling, learning, and testing processes during the implementation of the automatic form filling module in the field of internal medicine
DBCB2	66 text conversations with chatbot component for pediatrics	Data used in labeling, learning, and testing processes during the implementation of the automatic form filling module in the field of pediatrics

### Transcription and annotation processes

Anonymised data collected in the DBMI1, DBMI2, DBVB1, and DBVB2 databases were subjected to manual and automatic transcription processes, resulting in complete transcripts of patient-physician conversations presented in the form of the DBT1, DBT2, DBVT1, and DBVT2 databases, respectively. Manual (reference) transcriptions helped verify the effectiveness of the automatic transcription method used. Based on the transcripts, an annotation process was carried out, resulting in the creation of sets of labels identifying the following categories: medical symptom (MS), medicine’s name (MN), numerical data (ND), referral (R). Dialogues have been divided into phrases so that each phrase contains no less than two and no more than five medical words. Description of the created set of labels is provided in Table 5.

**Table 5:** Division of the label set into categories with examples.

Category	Symbol	Number of label	Sample labels
Medical symptom	MS	*INT 235* *PED 330*	Mouth ulcers, angina, insomnia, diarrhea, sore throat, back pain, earache, chills, fever, hearing loss, nausea, fainting, liquid stools, heart murmurs, insect bites, enlarged lymph nodes in the neck, heart rhythm disturbances, skin changes, degeneration of the spine
Medicine’s name	MN	*979 INT * *534 PED*	Acc, Amotaks, Apap, Augumentin, Buventol, Ceroxim, Dicortineff, Erdomed, Nebbud, Ospamox, Ventolin, Trilac, Zinnat
Numerical data	ND	*INT 6* *PED 6*	Weight, height, age, body temperature, blood glucose, cholesterol
Referral	R	*INT 62* *PED 46*	Referral to a surgeon, referral to an ophthalmologist, referral for a glucose test, referral for an ALT test, referral for an ultrasound, referral to a sanatorium

*INT* internal medicine, *PED* pediatrics.

**Fig. 1:**
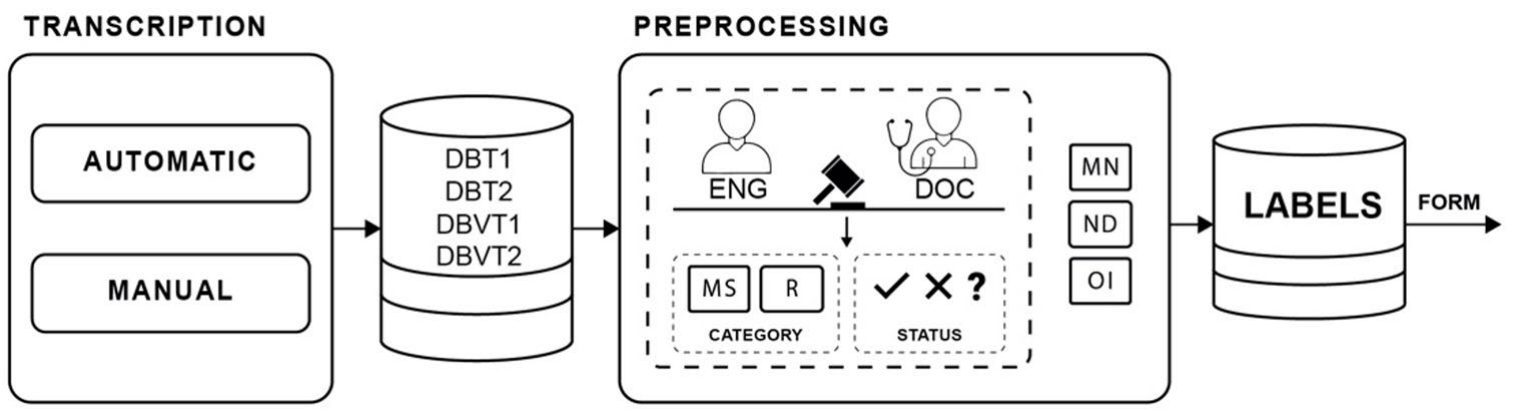
Data processing, where: *ENG* engineer, *DOC* physician.

A total of 1757 unique labels were identified, including: in the MS category—398, MN—1280, ND—6 and R—73. All other phrases in the conversations were assigned as category OI — other information. Assigning the appropriate labels to selected phrases containing individual statements is a rather difficult and time-consuming process. Therefore, a total of three people were involved in the annotation process, including two algorithm design engineers and (depending on the speciality) an internist or paediatrician. In problematic cases, questionable labels were consulted within a three-person panel, and the final decision was taken by vote. During the work on the sets identifying MS and R categories, status information was also supplemented simultaneously. The following statuses were established: Yes (Y), Question (Q) and No (N). The Yes status was assigned to phrases where confirmation for a given label was identified, the Question status was assigned to phrases where the interlocutor posed a question about a given label, while the No status was assigned when a given label was negated by the interlocutors. Ultimately, data for which there was complete agreement among annotators was included in the pool. The next step was to divide the statements into those containing names of medication names, numerical and other data. A subset with numerical data is used to determine a patient’s age, weight, height, body temperature or blood pressure. The labels are directed to the method that automatically fills in the form in the section concerning the interview conducted by the physician with the patient. They also provide input for the expert system. The process of data processing is illustrated in Fig. 1.

### Obtaining information from medical interviews

The method for automatically extracting information from medical interviews used a generative language model pre-trained on a Polish language corpus called plT5^30^. Its significantadvantage from the point of view of the problem described is that it analyses speech context and can be further tuned to classify medical dialogues. For example, Table 6 presents a dialogue where the physician replies “No, there is no need” (F5). This answer can have different meanings depending on the context. In the situation described, the physician explains that the patient does not need to perform a liver function test. Thus, no referral for testing will be made, and the label status in this case will be negative. Analysis of only single phrases without context would result in misclassification, as the patient’s statement (F2) includes information about the need for liver function tests, which without a full analysis of the dialogue could suggest the need for a referral for testing, resulting in a false-positive label. This indicates that the correct information about the label and its status contained in the dialogue is highly dependent on rich contextual information. The use of the T5 model makes it possible to solve such problems. The semantic extraction task involved the automatic extraction from medical dialogues of information such as disease symptoms, disease names and referrals for examinations. In addition, for each extracted label, its semantic status was determined. The statuses were defined in three categories: confirmed, denied and unknown. The whole task was formulated as a multilabel and multiclass classification problem because a single phrase could contain multiple categories at the same time, and one of several possible statuses was assigned to each category. The model was implemented based on the Hugging Face Transformers (HFT) library^33^ and tuned based on the analysed transcripts using a sliding window of 2 to 4 phrases with a step equal to 1. Appropriate labels are assigned to each window, containing specific terms and their statuses. These labels serve as cues for generating the correct medical terms and their statuses, improving the textual semantics for better understanding of medical dialogue. The process of fine-tuning the plT5 language model involved dividing the data into three sets: training (65%), evaluation (18%) and testing (17%). The fine-tuning of the model was conducted for 50 epochs, and the model with the highest F1 score on the evaluation set was selected as the final model. The use of the plT5 model, therefore, allows the system not only to simply recognise individual words but, more importantly, to understand their semantic meaning in the context of the entire conversation. As indicated above, this enables the Parrot AI system described above to distinguish whether, for example, the doctor is issuing a referral for tests or merely discussing potential further tests with the patient.

**Table 6:** Context of statements.

Phrase	Person	Dialogue	Assigned label
F1:	PHYSICIAN	Have you had an abdominal ultrasound?	Referral to an ultrasound (Q) Referral to an ultrasound (Y) Referral to liver function tests (Q) Referral to liver function tests (N)
F2:	PATIENT	No, do we need to do an abdominal ultrasound? Last week, we were at the hospital and the physician said that liver function tests need to be done	
F3:	PHYSICIAN	These results look good. You can have an abdominal ultrasound.	
F4:	PATIENT	And do we need to repeat the liver function tests?	
F5:	PHYSICIAN	No, there is no need	

An important element of the developed solution is data postprocessing, which includes the process of normalising the detected entities. Due to the diverse vocabulary found in the interviews and the large number of medical terms used, it is necessary to standardise the identified expressions. This process involves detecting equivalent expressions and reducing them to canonical form, which allows correct mapping of semantic references and minimising errors due to the variety of linguistic forms used by interlocutors.

### Diagnosis and therapy prediction system

In the case of the expert system, a multi-module architecture consisting of three main components was used: a data processing module, a diagnosis and therapy modeling module, and a result interpretation module. The data processing module, using normalisation, mapping and validation techniques, performs a multi-step process to prepare input labels. The system uses JSON structures to effectively categorise medical diseases according to the ICD-10 classification. The following algorithms were implemented in the diagnosis and therapy modeling module: Decision Tree, Naive Bayes, KNN, SVM, Random Forest, and XGBoost. The choice of this group of classifiers was dictated by their best performance obtained during the research work. From the perspective of the system’s operation, these algorithms are used to automatically generate prompts for the doctor to suggest the most likely diagnosis. The developed models use previously identified symptoms as input data. Detailed test results of each classification method are summarised in “Expert system”. The results interpretation module, in turn, implements a set of quality control mechanisms for the output data. The convergence of the system’s proposed diagnoses and therapies with the medical knowledge contained in the knowledge base is checked. In addition, formal validation is performed, which includes validation of the data structures sent to the form input. The three most likely suggestions for diagnosis proposed by the system are displayed as a list in the doctor’s panel. For each item, the percentage probability of a given diagnosis is determined. In the next step, the doctor assesses and validates the plausibility of the suggestions offered by the system. The final decision always rests with the specialist: the doctor can accept, modify or completely reject every recommendation of the system. This way, on the one hand, automation supports the processes performed by doctors, while on the other hand, the risk of errors that could affect patient safety is effectively minimised. In addition, all of the doctor’s interactions with the system are continuously monitored, guaranteeing the auditability of decision-making processes and allowing full transparency in the implementation and ongoing monitoring of AI-based tools.

### Contact center system components

CC system components in the form of virtual intelligent assistants—voicebot and chatbot—have also been integrated into the system to carry out the registration process and collect data from the initial interview. In the case of a medical facility, the integration of these components has enabled the remote registration of patients while collecting key data on their health status even before their scheduled visit. This saves time for both the doctor and the patient during the eventual medical visit. In addition, these solutions relieve the staff at the clinic from routine, repetitive duties. The FreeSWITCH telecommunications server was used for the voicebot, offering a modular architecture that allows for easy expansion of individual functionalities. It supports SIP/RTP (Session Initiation Protocol/Real-time Transport Protocol) technologies, which ensure real-time operation. The words spoken by the caller are sent directly to the speech transcription module, where a transcript is created, which is then processed by the conversation platform. This platform is responsible for recognising the patient’s intentions. The next step is to generate the response text, which is sent to the TTS (Text-To-Speech) algorithm. In case of difficulties in recognising the intention, the call is redirected directly to the registration operator at the clinic. Appropriate mock-ups with graphic elements were created for the chatbot module, taking into account current trends in UX/UI usability principles. The interface for mobile devices has been adapted to Responsive Web Design (RWD) principles. Communication through popular communication platforms has also been made possible. The frontend of both solutions was implemented using the Angular framework, while the backend was implemented using SpringBoot. Detailed results foreach component are summarised in “CC system components”.

## Proposed approach

The proposed system has been developed as a hybrid combination of various innovative solutions, creating a comprehensive, multimodal environment that supports the various tasks performed by general practitioners during standard patient appointments. The system structure comprises four main blocks, identified as follows: (a) CC system components managing voicebot and chatbot services, (b) components supporting transcription processes, (c) a proprietary solution for obtaining information from medical interviews, and (d) a proprietary expert system solution with a knowledge base. The system operates to meet real-time requirements.

**Fig. 2:**
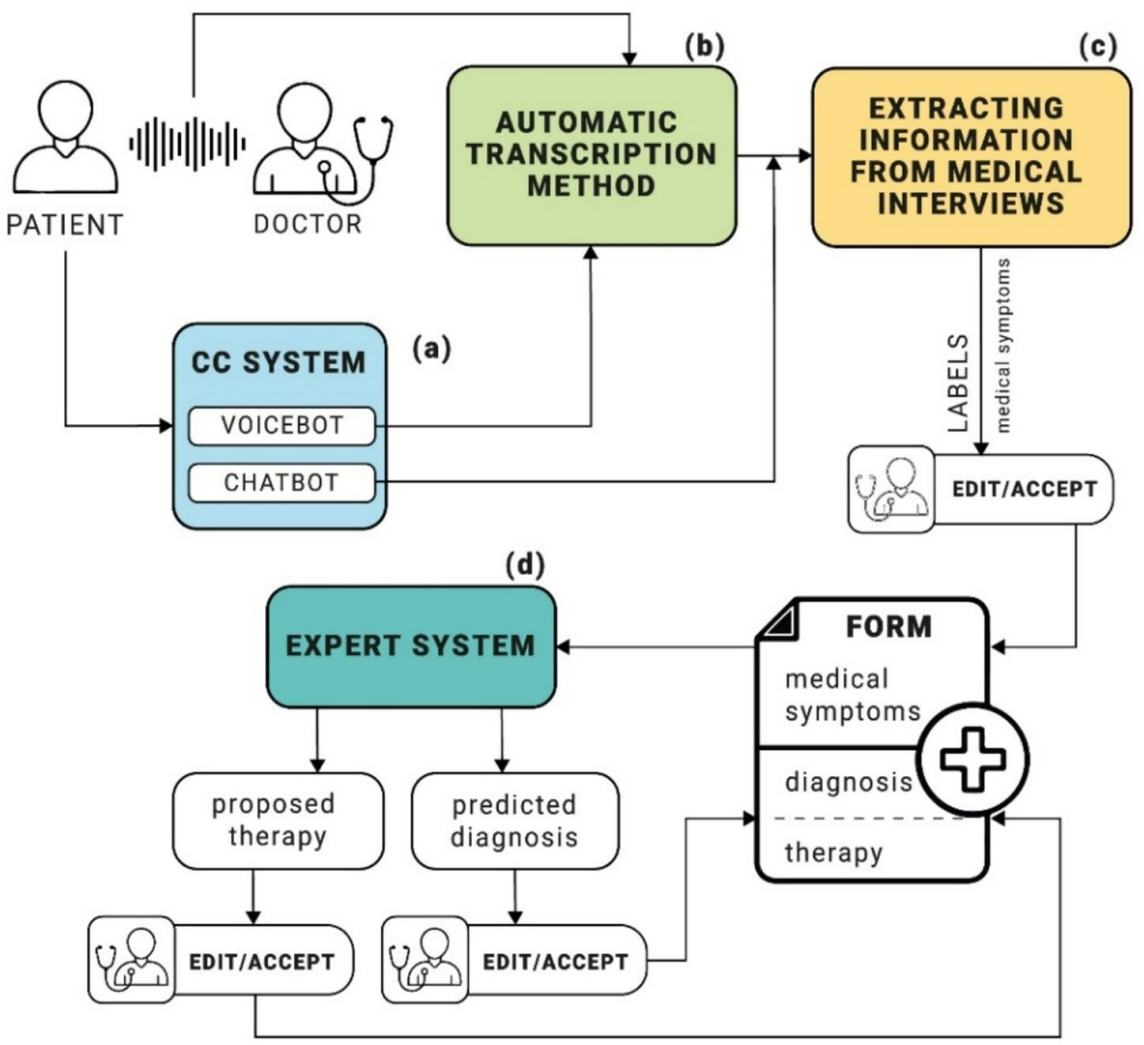
Parrot AI system supporting physicians’ work.

The proposed solution may start operating at two different stages. The first standard approach is for the physician to conduct a full classic interview directly in their office, while the second option offers support for medical staff through voicebots or chatbots implemented in CC-class systems. Traditionally, the physician has no prior knowledge about the patient prior to the appointment. In the second case, CC tools (a) support the registration process and are also used to collect preliminary information containing relevant data about the reason for the patient’s appointment. In the case of voice interaction, the conversation is transcribed using the ASR module (b), which is the first stage of voice data processing. As a result of its operation, individual statements are converted into text data, which forms the basis for further analysis. In the case of text (chat) interaction, the transcription process is not necessary. The text data obtained is transferred to a method that automates the process of extracting information from medical interviews (c). The result is a partially completed medical form, which is then presented to the physician for editing and/or approval. Data from the verified first part of the medical form is then transferred to the expert system (d), which recommends a proposed diagnosis based on the information provided. The proposed diagnosis is also subject to editing and/or approval by a physician, after which the system suggests appropriate treatment in the form of recommendations for recommended medications and potential referrals and sick leave. These results also require final verification, editing, and/or approval by a physician. The expert system automates the tasks related to filling out the second part of the medical form. A diagram illustrating the principle of operation is presented in Fig. 2.

A summary of the core technologies and frameworks used to implement the Parrot AI system is provided in Table 7. It features a description of the elements related to the design of the system architecture and the method of communication between individual blocks. In addition, it indicates the basic technologies used in the preparation of individual system components.

**Table 7:** Technologies used in the Parrot AI system.

Block	Technology	Description
*Architecture and communication*		
Frontend	Angular	The choice of this technology was influenced, i.a., by: (a) component-based architecture, which facilitates the creation of scalable applications; (b) two-way data binding, ensuring synchronization between the model and the display; (c) dependency injection, which improves modularity and code testing capabilities; (d) an extensive ecosystem of tools
Backend	Spring boot	A Java framework that simplifies the creation of applications based on Spring technology. The choice of this technology was influenced, i.a., by: (a) automatic configuration, which reduces the need for manual work, (b) a built-in application server, eliminating the need for external deployment, (c) starter dependencies, which simplify dependency management, (d) actuator, providing ready-to-use monitoring and management functions
Database	PostgreSQL	Relational database management system. The choice of this technology was influenced, i.a., by: (a) compliance with the SQL standard and JSON support, (b) advanced data types, including geometric and network types, (c) extensibility through custom data types, functions, and procedural languages, (d) support for ACID transactions, ensuring data integrity
Buffer layer	Redis	A key-value database operating in the operating memory. The choice of this technology was influenced, i.a., by: (a) high performance resulting from data storage in RAM, (b) diverse data structures, such as strings, hashes, lists, sets, (c) data persistence mechanisms through disk storage, (d) support for master-slave replication and clustering
Communication	REST API, RabbitMQ	The choice was based on the need to support two types of communication: (a) REST API for synchronous communication due to its statelessness and uniform interface; (b) RabbitMQ for asynchronous communication, ensuring reliable message delivery and flexible queuing
*Selected components*		
CC system	FreeSWITCH, TTS, STT, Bot	Components used by the voicebot and/or chatbot necessary to handle user interactions in automatic registration processes and during initial interviews
ASR method	Transcription method based on the Whisper Large engine – v3	A solution developed by the authors specifically for applications in the medical industry
NLP analysis	PyTorch DDP, plT5 model, AdamW optimizer	Technologies used in the method developed by the authors, which automates the process of extracting information from medical interviews
Expert system	Scikit-learn, Pandas, NumPy, Optuna optimizer	Selected technologies ensure: (a) high-speed numerical calculations; (b) effective data preparation and cleaning; (c) access to key classification algorithms in Scikit-learn; c) automated model tuning thanks to

*ACID* atomicity, consistency, isolation, durability, *TTS* text- to speech, *STT* speech to text, *PyTorch DDP* PyTorch distributed data paralle

**Fig. 3:**
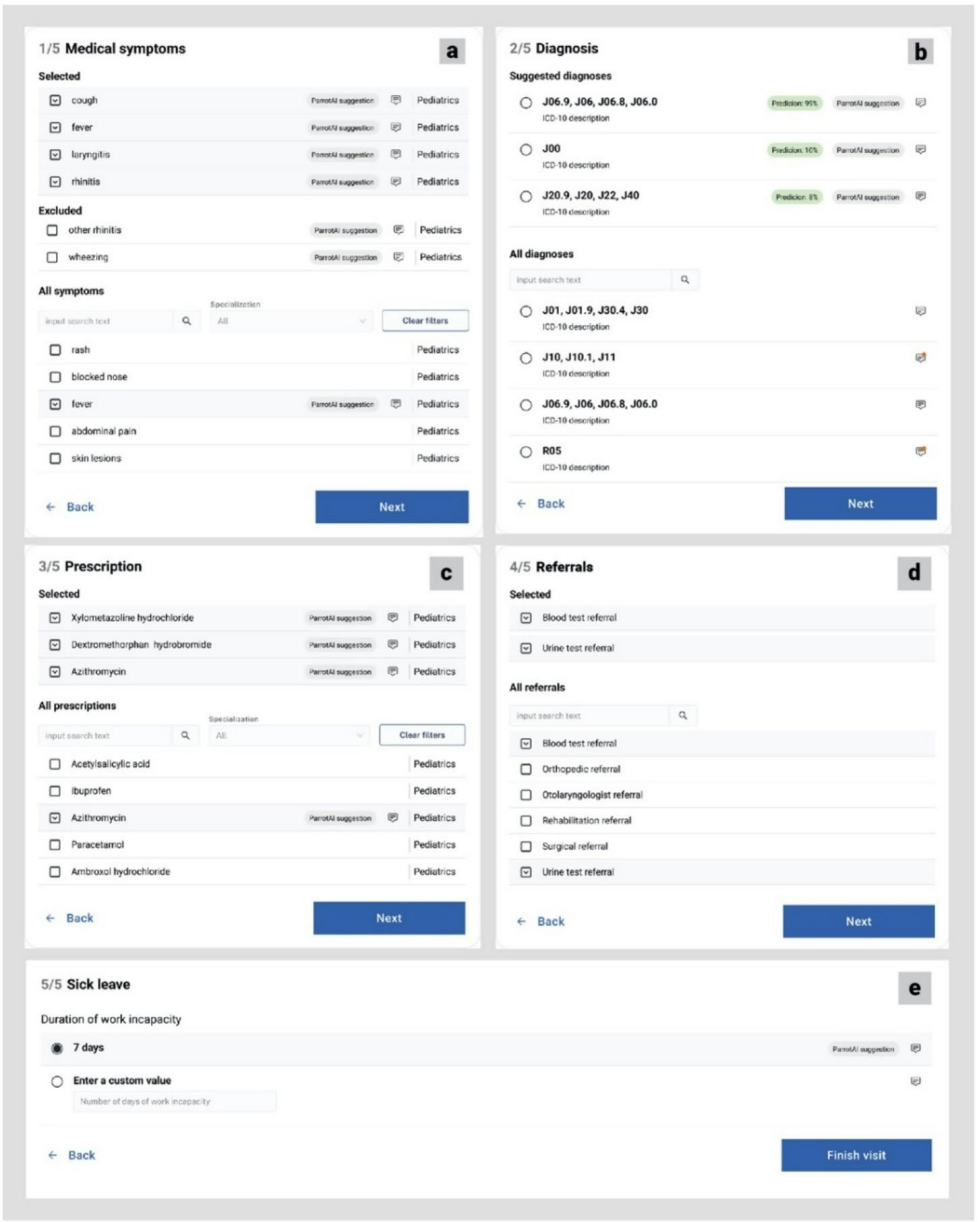
Parrot AI user interface illustrating the automated form filling process **a** medicalsymptoms; **b** diagnosis; **c** prescription; **d** referrals; **e** sick leave.

Figure 3 presents the user interface for the Parrot AI system, illustrating the automated form filling process. In line with the method described above, the system guides the physician through a structured process aimed at quickly and accurately converting the patient interview into the target form.

**Fig. 4:**
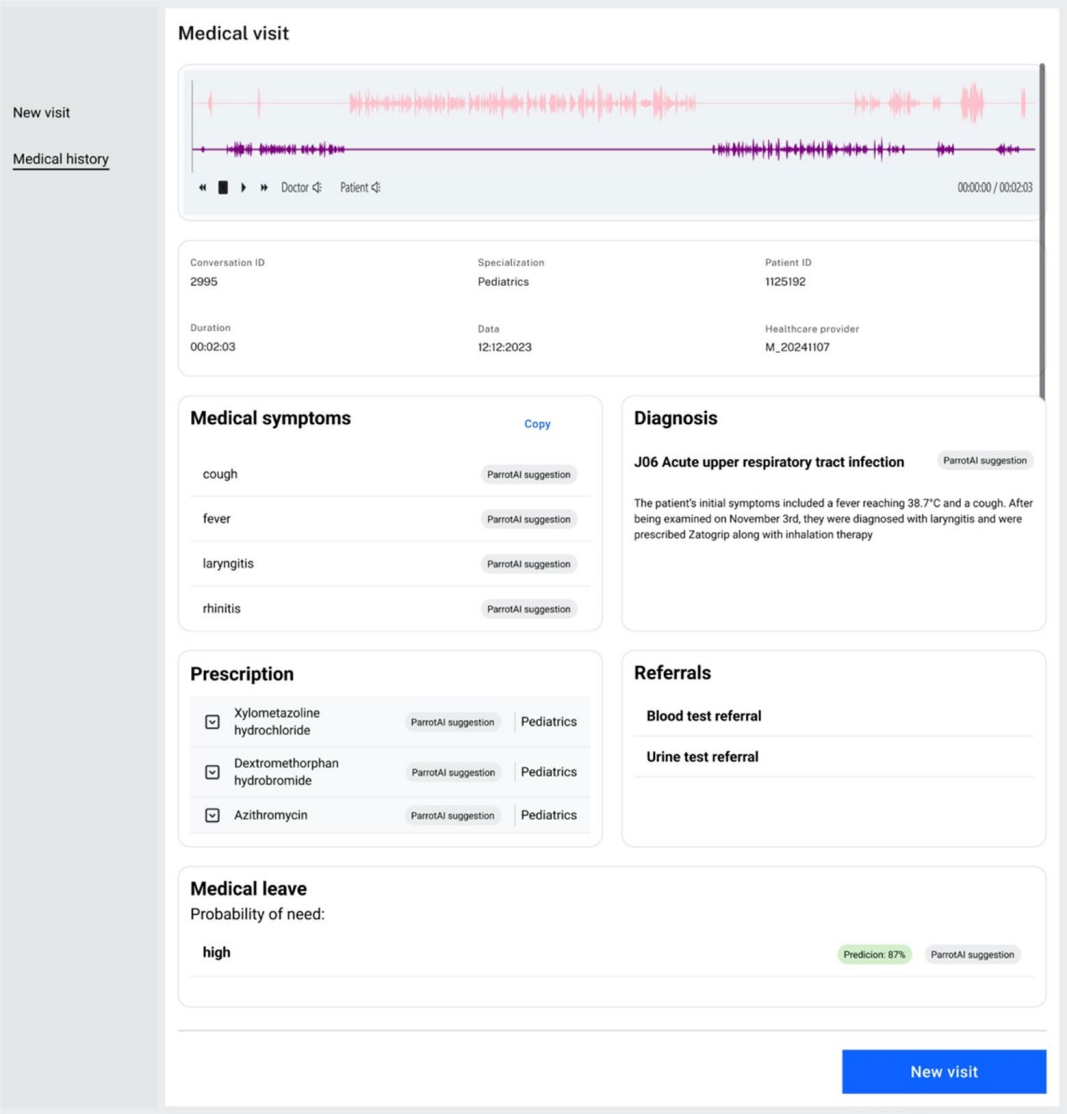
Summary view of the patient’s appointment in the Parrot AI system.

The final result of the system’s functionality is a summary of the patient’s appointment, as shown in Fig. 4. This view aggregates all key information generated during the appointment and approved by the physician.

The system originally operates in Polish, although, for the purposes of this article, individual views have been translated into English. The next section presents the research findings.

## Results

This section presents the results of tests for individual modules of the developed Parrot AI system.

### Semantic analysis of medical interviews

In step one, a comparative study of the effectiveness of extracting medical symptoms and assigning them semantic statuses using the plT5 model was conducted with other Polish language models in the base version. The evaluation was performed on randomly selected test sets derived from a collection of medical dialogues concerning internal diseases. Each collection comprised approximately 140 complete dialogues. The plT5^34^ model performed best, with *Precision = 83.17%*,* Recall = 80.12%* and *F1-score = 81.62%*, indicating its high effectiveness both in identifying medical entities and determining their statuses. Another generative model, plBART^35^ produced moderately satisfactory results: *Precision = 74.75%*,* Recall = 71.08%* and *F1-score = 72.87%*, whereas models based on the BERT architecture scored significantly lower – PolBERT^36^ (*Precision = 53.62%*,* Recall = 67.75%* and *F1-score = 59.86%*) and plRoBERTa^37^ (*Precision = 62.48%*,* Recall = 35.78%* and *F1-score = 45.50%*). It can be seen that generative models are more effective with sequence extraction in Polish than encoder models, which showed a greater imbalance between precision and sensitivity. Based on these results, the plT5 model was selected for further experiments.

Detailed results of the study demonstrating the effectiveness of the method of automatic information extraction from medical interviews for both specialisations are presented in Table 8. The reported metrics (*Precision*, *Recall*, *F1-score*) relate directly to the dialogue-level. This way, true positive is defined as the correct identification of a medical entity (e.g., a symptom) and its status within the entire medical history conversation – regardless of how many times or in which minute of the conversation the information appeared. The final results were obtained after conducting a cross-validation process with a multiplicity of k = 6. The AdamW optimiser^38^ was used, and the weight drop was set to 0.01. The initial learning rate was set to 2e^–5^, the batch size parameter was 4, and the total number of epochs was estimated at 50. A greedy search algorithm was applied.

**Table 8:** Results of semantic analysis of interviews.

*k*	Internal medicine specialist			Pediatrics specialist		
	Precision (%)	Recall (%)	F1-score (%)	Precision (%)	Recall (%)	F1-score (%)
1	81.59	84.52	82.45	81.07	85.03	81.63
2	83.09	82.90	82.65	81.09	83.60	82.00
3	82.54	81.98	81.80	85.87	89.81	87.42
4	81.47	85.61	83.19	80.30	84.36	81.92
5	87.59	89.28	89.62	84.58	89.58	86.76
6	83.97	88.48	85.79	81.87	84.52	82.81
Avr (%)	83.38	85.96	84.25	82.46	85.82	83.76
Std (%)	2.26	2.94	2.97	2.23	2.78	2.62

*Av* average value, *Std* standard deviation.

The average effectiveness scores for automatic form field completion in the section concerning the scope of medical interviews are given in line *Avr*. In all cases, the standard deviation did not exceed 3%, which indicates that the tested models are fairly stable. Consistency across different folds demonstrates the robustness of the plT5 model in processing diverse medical dialogues. It can be seen that the differences between the models are insignificant, but the noticeable advantage of *Recall* over *Precision* suggests that the system effectively identifies the vast majority of correct labels, while generating a certain number of additional labels that are not found in the reference data set.

**Fig. 5:**
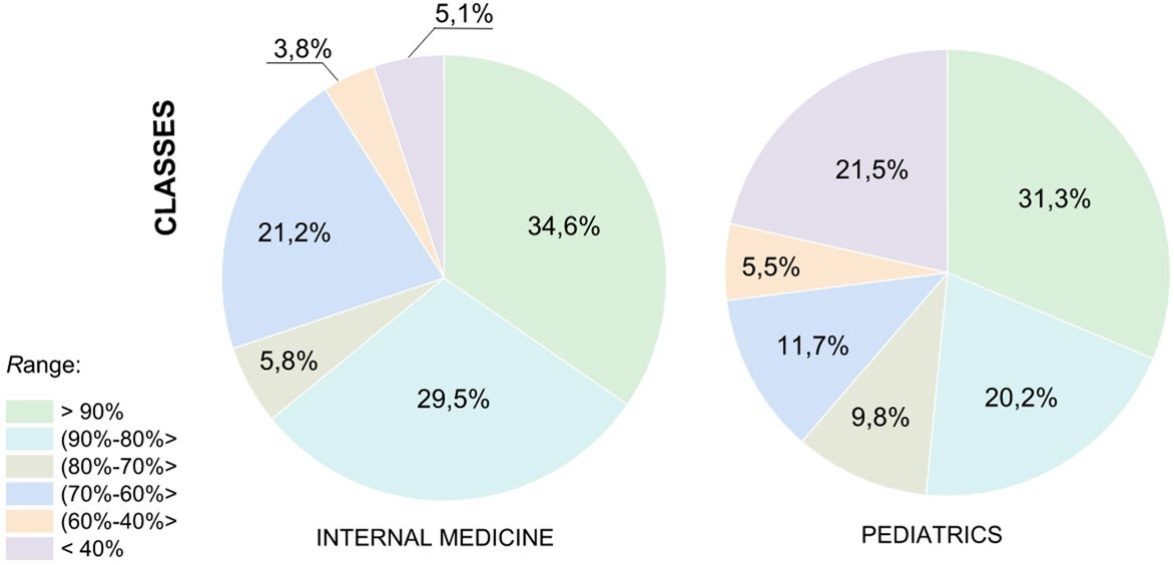
Distribution of the number of classes depending on the F1-score value.

**Fig. 6:**
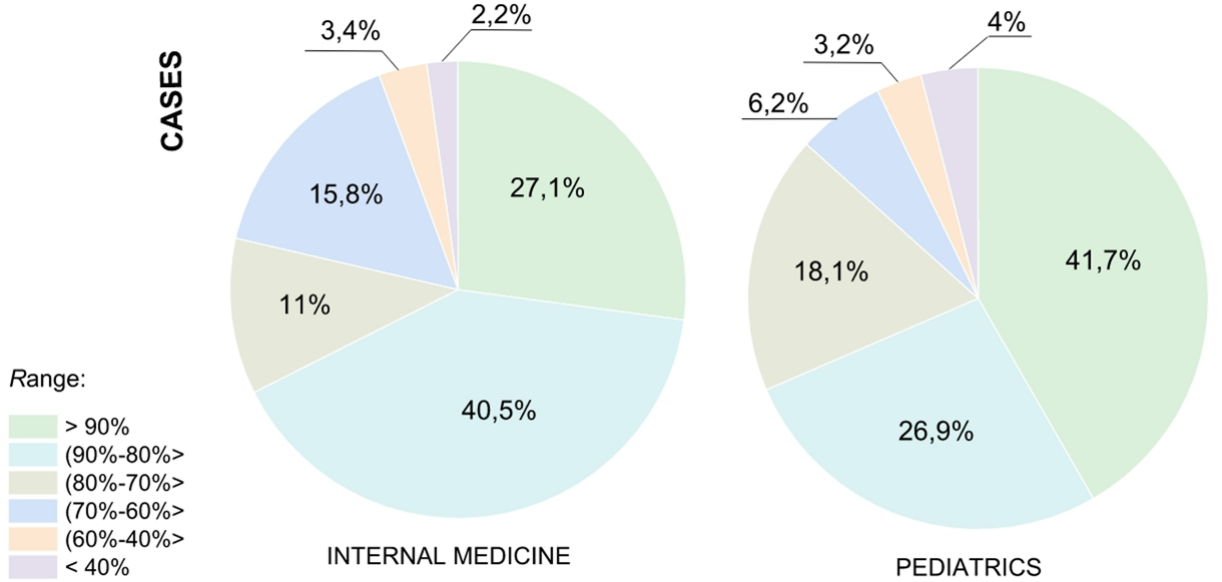
Distribution of the number of cases depending on the F1-score value.

Figures 5 and 6 illustrate the effectiveness of extracting symptoms from medical dialogues based on test sets. Classes correspond to medical symptoms, while cases refer to individual medical interviews. The results presented only concern symptoms with a positive status, as these play the most important role in subsequent stages of the system’s operation. Classes corresponding to referrals for tests were also omitted. Figure 5 shows the distribution of classes by F1-score values ​​for pediatrics and internal medicine, respectively. Figure 6 shows the distribution of cases by F1-score values.

The analysis of the medical symptom class distribution shows that for internal medicine (Fig. 5, left chart), the largest group (34.6%) was classified with the highest performance (F1 > 0.9). For pediatrics (Fig. 5, right chart), the distribution was more varied, with 31.3% classed in the highest performance threshold. As was observed, well-detected symptoms were those with a short, unambiguous definition (e.g., bruises), whereas low performance medical symptoms were characterised by complex descriptions (e.g., mucopurulent discharge in the eyes).

Figure 6 shows how these results translate to the number of actual dialogues. In the case of internal medicine (Fig. 6, left chart), as many as 40.5% of all dialogues concerned medical symptoms that the model recognises with the highest performance (F1 > 0.9). A similar trend was observed for pediatrics (Fig. 6, right chart), where 41.7% of dialogues belonged to the same, most effectively recognised group.

### Expert system

Detailed research results showing the effectiveness of the developed expert system in predicting diagnoses and proposing specific therapies for both specialisations are presented in Table 9. The Optuna optimiser^37^ was used to automatically adjust model parameters.

**Table 9:** Results of expert system testing.

Method	Internal medicine specialist				Pediatrics specialist			
	Accuracy (%)	Precision (%)	Recall (%)	F1-score (%)	Accuracy (%)	Precision (%)	Recall (%)	F1-score (%)
RF	**87.50**	**89.32**	**87.50**	**87.62**	**84.58**	**85.55**	**84.58**	**84.35**
KNN	84.06	86.85	84.06	84.12	80.84	83.37	80.84	81.40
SVM	81.88	83.87	81.88	81.77	83.98	85.17	83.98	83.84
XGBoost	78.44	81.20	78.44	78.53	76.02	79.40	76.02	76.98
NB	55.80	68.81	55.80	54.10	37.59	48.94	37.59	36.49
DT	46.92	62.02	46.92	49.60	45.30	50.12	45.30	46.36

*RF* random forest, *NB* Naive Bayes, *DT* decision tree.

In terms of the ability to predict medical diagnoses for both specialisations, the Random Forest model proved to be the most effective. The Random Forest classifier achieved the highest F1-score (87.62% for internal medicine and 84.35 for pediatrics), significantly outperforming baseline models like Naive Bayes, which confirms its suitability for complex medical decision-making tasks. Existing expert systems typically achieve a diagnostic accuracy of 70–80%, whereas we scored more than 84%. The results confirm that the proposed method really works.

**Fig. 7:**
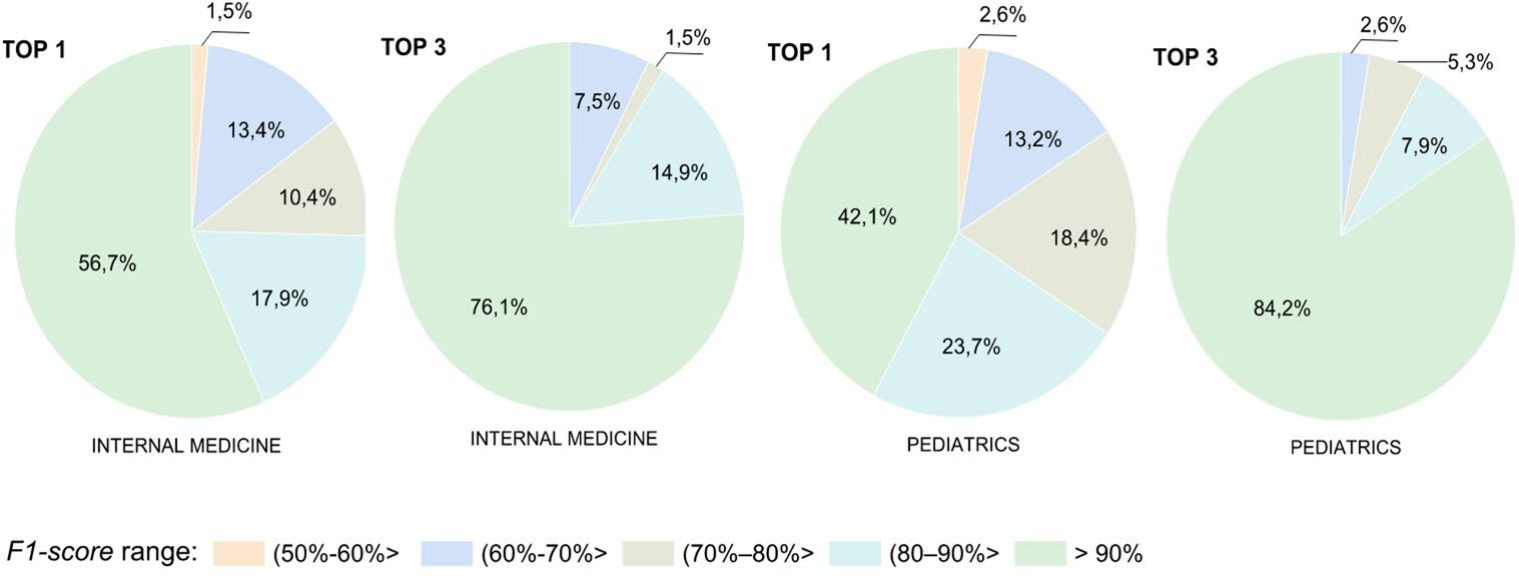
The effectiveness of diagnosis prediction determined by the f1-score metric, obtained for internal medicine and pediatrics.

Figure 7 presents the average prediction efficiency, measured by the F1-score, for each type of disease identified. The results refer to the RF classifier, as it yields the best results, as shown in Table 9.

It can be seen that in the case of predicting the diagnosis for TOP 1, where only the first model suggestion is taken into account, a significant scatter of results was obtained. In the case of internal medicine, 56.7% of disease entities were classified with the highest accuracy. For pediatrics, the rate was 42.1%. Significantly better results were obtained for TOP 3. In both specialties, for the vast majority of diseases, the model achieved the highest accuracy threshold – this was true for 76.1% of cases in internal medicine and as many as 84.2% in pediatrics. This confirms the model’s ability to significantly narrow down the list of potential diagnoses, providing physicians with real support.

### CC system components

As part of the tests, ten independent participants conducted a total of 125 voice calls with the voicebot and 142 text conversations with the chatbot. Each tester completed at least five interactions for internal medicine and pediatrics in both components. Detailed results are presented in Table 10. In all cases, a very high FCR (First Call Resolution) was achieved, confirming that the bots are highly effective.

**Table 10:** Results of CC system testing.

Component	Internal medicine specialist			Pediatrics specialist		
	Number of testers (pieces)	Number of tests (pieces)	FCR (%)	Number of testers (pieces)	Number of tests (pieces)	FCR (%)
Voicebot	10	64	85	10	61	83
Chatbot	10	76	82	10	66	81

Interactions with the voicebot and chatbot were examined using a semantic analysis method developed for medical interviews. This process enabled the medical form to be partially completed based on symptoms identified in the initial interview. This method demonstrated strong performance on the data collected by the bots, with 93.53% precision, 86.24% recall, and an F1-score of 88.76%. These findings highlight the feasibility of applying conversational computing (CC) solutions to the automation of medical processes.

## Discussion

The research presented in the individual paragraphs of “Results” reveals that machine classification for all components developed by the authors ranges between 82.46% and 89.32% in terms of effectiveness. When analysing the efficacy of a number of medical systems currently in use, it can be observed that they achieve similar results. For example, a system dedicated to generating medical reports^40^ achieves a declared effectiveness of F1 = 72%. A system supporting the diagnosis of glaucoma^39^ achieves an accuracy of 83.4%, while a system supporting the coding of medical procedures^42^ demonstrated Accuracy = 87.08% and F1 = 85.82%. The paper^43^ presents an intelligent chatbot used to simulate conversations with patients, which achieved the highest diagnostic accuracy of 81.8%. Unlike most existing solutions, which, as indicated, focus only on selected tasks (e.g., report generation, diagnosis support, medical procedure coding, or simulations of conversations with patients using chatbots and/or voicebots), the proposed Parrot AI system is the first to provide full support for the entire course of an appointment by combining multiple dedicated components. The knowledge gained through machine identification of the patient’s symptoms mentioned during the interview at the beginning of the appointment is particularly important in terms of automatically filling out the first part of the medical form. The knowledge generated by the expert system, on the other hand, assists in completing the second part. In addition, as is well known, virtual assistants have become increasingly common in CC systems, replacing humans in solving problems of increasing complexity^44^. In the case of the Parrot AI system, patients can use avoicebot or chatbot to make an appointment or report initial symptoms. The information collected in this way is then used during the appointment with a physician, where the system continues to collect data and helps structure the medical interview. Taking into account the role of bots can therefore significantly reduce the length of appointments, as much of the information is already obtained by the physician before the appointment begins. Therefore, in order to effectively assist physicians in automating the process of filling out a complete form, the three above-mentioned components must be integrated. The proposed approach not only speeds up the process of creating EHRs, but also significantly reduces the risk of error from manual data entry. At the same time, the results in terms of system effectiveness presented by other researchers, which are similar to those obtained for the Parrot AI system, directly indicate its practical application potential.

Compared to existing physician support solutions, the Parrot AI system automates all stages of the patient visit by transcribing and documenting the visit registration process, transcribing and documenting the patient’s visit, extracting information about symptoms, diagnosing the patient’s illness and providing suggestions for recommended treatment, referrals for tests and medical leave. Existing systems, despite their advanced capabilities, only support selected stages of the patient’s visit. The international systems Nabla^16^ or Dragon Medical One^19^ transcribe medical history conversations and support the creation of documentation, but they do not support decision-making. Infermedica^18^ or IBM Watson Health [20] support medical diagnosis but do not enable the documentation of the patient visit and extraction of symptoms from the medical history conversation. None of the systems mentioned automate the process of visit registration. Also, systems available only for Polish perform only selected tasks: such as transcription and generation of notes from the visit (Noa^21^) or pre-visit (Previsit^22^), registration of visits (Talkie AI^23^) or transcription and documentation of the visit (ADMED VOICE^24^). The developed system is not just an integration of existing solutions. The transcription method used in the system, the semantic analysis of the medical history conversation, and the expert system are proprietary solutions based on artificial intelligence and machine learning. The results obtained for real recordings of medical history conversations confirmed the high quality and effectiveness of the developed methods.

The limitations of this study are typical for research on medical systems. The following points present the possible limitations identified by the authors, which may also constitute potential areas for further research:

- A major limitation of this study is its linguistic scope. The results are based on data collected in Polish, meaning the current system cannot analyse interviews in other languages. Consequently, the dataset is not fully representative, and adapting this solution would require new data and fine-tuning with language-specific models. The data were also drawn from facilities in close proximity, so regional variation in dialects, colloquialisms, and accentuation may further affect performance. These limitations impact the universality of the system, which is a common challenge in the clinical implementation of AI solutions^45^. Therefore, continuous monitoring of the solution’s performance is essential to enable further retraining and positively influence the system’s universality.
- A further limitation concerns the demographic context. The findings reported in this study are derived from data collected within a restricted geographic area, which limits the generalisability of the results to broader populations. For example, tropical diseases are generally absent in Central European populations. This represents a considerable challenge for the international deployment of the system and underscores the the need to adapt and validate it using region-specific clinical datasets.
- It should be noted that the system was trained to recognise disease entities directly identified in the collected interviews. Although the dataset was relatively extensive, it is possible that not all clinical cases were covered while preparing the data. Consequently, the system may not accurately predict diagnoses for diseases not represented in the training data. This highlights the need for a systematic expansion of the knowledge base to support further development of the system.
- The authors’ experience suggests that during interviews conducted in real-world medical settings, ambient noise may substantially affect the performance of the developed system. While high-quality equipment was used during the study, practical deployment requires addressing acoustic optimization in clinical environments. Ensuring high transcription precision under dynamic conditions remains a priority, as lower-specification microphones or elevated background noise could degrade system performance. This underscores the need for further research to identify optimal environmental conditions for implementation in individual medical facilities.
- Interview samples were collected from the two specialties outlined above (internal medicine and pediatrics). The selection of these specialties was guided by the social, economic, and market considerations discussed in “Introduction”. Consequently, the generalisability of the results to other medical specialties remains uncertain. Addressing this limitation in future research would require access to specialised datasets, which are often difficult to obtain in medical contexts, particularly for less widely spoken languages^46^.
- To date, the system has been evaluated only under laboratory conditions using historical clinical data, which may not fully represent the complexities of routine clinical practice. The transition from laboratory-validated results to real-time clinical environments limits the assessment of the system’s robustness to unforeseen scenarios. It should be noted that the system is currently in a testing phase conducted directly in medical offices, and the preliminary results obtained are similar to those presented in this work. Nevertheless, these aspects will be the subject of further detailed research in subsequent phases of system development.
- The implementation of a system in real-world clinical settings presents significant challenges at multiple levels. These include ensuring compatibility with existing IT infrastructure, providing adequate computational resources, and maintaining secure data transmission and processing^45^. Beyond technological considerations, legal and ethical requirements are critical for AI-based solutions and should be addressed throughout the design, implementation, and operational phases^48^. Particular attention must be paid to the protection of sensitive information, including personal data and research outcomes, which is regulated differently across countries and may necessitate additional preparatory measures depending on the geographic location of the medical facility^47^. Moreover, user awareness and acceptance—among both physicians and patients—are essential, as resistance to new technologies may hinder their routine adoption in clinical practice^48^.

## Conclusion

In this paper, a system for automating medical processes, such as filling out medical records and supporting medical decisions, was presented. The proposed approach integrates (1) semantic analysis methods that use ASR, NLP, and generative AI/ML models to extract information from medical interviews; (2) a knowledge-based expert system that supports physician decision-making processes; and (3) CC components, such as virtual assistants for voice and text communication. By combining these components, the system provides a feasible foundation for the further development of smart medical systems that streamline clinical processes while maintaining the key role of the physician. The average results, obtained from real data with effectiveness across individual metrics ranging from 82.46% to 89.32%, suggest a significant potential for practical implementation.

Further research focuses on ongoing clinical tests in primary care settings, the preliminary results of which show a high consistency with the results presented in this paper. The completion of this phase of work will enable the additional verification of the effectiveness and usability of the system in the real conditions of everyday medical practice. The implementation of the Parrot AI system was designed based on the human-in-the-loop principle^49^, which had been recognised as the basis for building trust in the medical environment. By providing transparent suggestions that always require authorization from medical personnel, full control over the AI mechanisms is maintained. This approach ensures that final diagnostic responsibility always rests with the expert, which is critical for the successful implementation of AI-basedsolutions in medical facilities. However, the use of machine learning based on neural networks poses the so-called “black box” problem^50^. The development and application of explainability mechanisms for the AI methods used represent directions for further system development. Key operational challenges, such as the selection of specialised directional microphones and the reduction of ambient noise in doctors’ offices, are also taken into account in the study. In this respect, maintaining high transcription precision requires optimising the acoustic environment, including the strategic placement of directional microphones to minimise ambient noise and hardware-generated interference. This allows high transcription precision to be maintained under dynamic operating conditions, thus overcoming one of the limitations of the system described earlier. In terms of technical integration, the system is designed to work with existing infrastructure and other medical platforms using the REST API standard. The second area of development will be the expansion of the system’s functionality with additional medical specialisations, which will extend its application to a wider range of areas. The third direction could be the potential implementation of corrective mechanisms based on reinforcement learning algorithms, which would increase reliability and reduce the impact of erroneous or ambiguous input data. Future research should also evaluate not only the technical aspects of the system’s operation, but also its impact on patient satisfaction, staff workload, and the quality of medical decisions.

In summary, the presented system is aligned with the objectives of the digital transformation of healthcare worldwide. By combining automation, natural language processing and clinical decision support modules, it provides the basis for creating efficient solutions, while maintaining the key role of the doctor in diagnostic and therapeutic processes.

## Data Availability

The data for the described research, which forms the basis for the results of this article, is provided directly by the leader of the PARROT AI project - the company Altar Sp. z o. o.
